# Uphill or downhill bleeding?

**DOI:** 10.1136/gutjnl-2020-322298

**Published:** 2020-08-21

**Authors:** Zillah Cargill, Tamsin Cargill, Brian Lei, Noor Bekkali, James East, Jonathan Marshall

**Affiliations:** 1 Institute of Liver Disease, King's College Hospital NHS Foundation Trust, London, UK; 2 Translational Gastroenterology Unit, University of Oxford, Oxford, UK; 3 Horton General Hospital, Oxford University Hospitals NHS Trust, Banbury, UK

**Keywords:** oesophageal varices

## Introduction

An 87-year-old woman presented with a 2-day history of melaena and symptomatic anaemia. Medical history included idiopathic hyperthyroidism, a gastric ulcer and diverticular disease. On arrival, vital signs were stable apart from a tachycardia (107 bpm). Her initial haemoglobin was 48 g/L and urea 32.7 mmol/L. A large anterior neck mass was observed on examination.

**Figure 1:**
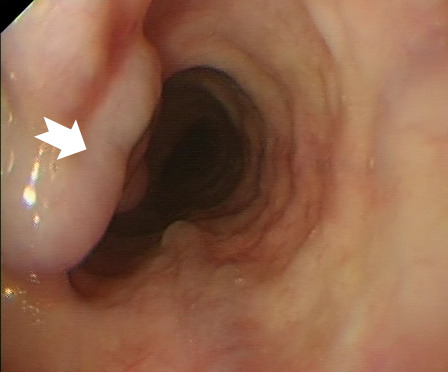
Endoscopic view of upper third of oesophagus.

**Figure 2:**
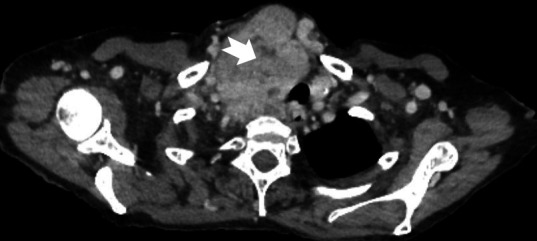
Axial CT image at the level of T3.

At endoscopy, this abnormality was identified in the upper oesophagus (figure 1). Minimal gastritis and a normal duodenum were also observed. Subsequently cross-sectional imaging of the chest abdomen and pelvis was undertaken to investigate the neck mass (figure 2).

## Question 1

What is the abnormality in the upper oesophagus and how should it be managed?

## Question 2

What is the significance of the right sided neck mass?

## Answer

**Figure 3:**
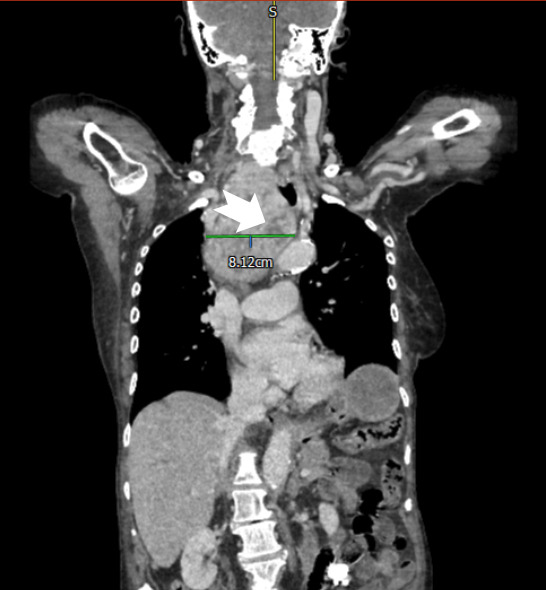
Coronal CT image of the chest and upper abdominal viscera showing a large left-sided irregular goitre (arrow) with normal appearance of the liver and spleen.

The patient had developed proximal oesophageal varices (figure 1) due to venous compression by a large goitre (figures 2 and 3 (arrow)). Grade 2 oesophageal varices were seen in the upper third of the oesophagus at the 11 o’clock position (figure 1 (arrow)) with no stigmata of recent bleeding and no red wale sign. The absence of portal hypertensive gastropathy at endoscopy, and the unusual variceal location suggests a cause other than portal hypertension. Additionally, no other features to suggest underlying portal hypertension or chronic liver disease were noted including a normal liver, spleen and absence of ascites on CT imaging (figure 3). The varices were managed without intervention with a strategy of watchful expectancy. Surgery, the definitive treatment of intrathoracic goitre, was declined by the patient.

Isolated proximal oesophageal varices are rare with an incidence of 0.5% of upper gastrointestinal endoscopies in a recently reported observational study.^1^ Development is often due to extrinsic processes including superior vena cava obstruction and mediastinal masses.^2–4^ Thyroid goitre as a cause has been reported but is uncommon.^1^

A variety of attempted treatment options for downhill variceal haemorrhage have been reviewed,^1^ including band-ligation and sclerotherapy. However, endoscopic treatment of haemorrhage can be difficult due to the anatomical location and a theoretically higher risk of post procedural bleeding or perforation.^5^

Evidenced-based guidelines are lacking, but non-selective beta blockers or band-ligation are not recommended as prophylaxis for preventing haemorrhage. Treatment of the underlying aetiology is the preferred choice. The low risk of haemorrhage from downhill varices, supports a conservative approach where there are no stigmata of bleeding at endoscopy.^1^

Endoscopists should be aware that identifying proximal varices at endoscopy should trigger follow-up investigations to find an underlying cause. A multidisciplinary review following identification of the aetiology could help to determine the best management strategy for these complex cases.
